# Neuropsychological Consequences of Chronic Drug Use: Relevance to Treatment Approaches

**DOI:** 10.3389/fpsyt.2015.00189

**Published:** 2016-01-15

**Authors:** Jean Lud Cadet, Veronica Bisagno

**Affiliations:** ^1^National Institute on Drug Abuse Intramural Program, Molecular Neuropsychiatry Research Branch, Baltimore, MD, USA; ^2^Instituto de Investigaciones Farmacológicas (ININFA), Universidad de Buenos Aires-CONICET, Buenos Aires, Argentina

**Keywords:** marijuana, cocaine, methamphetamine, frontal cortex, cognition

## Abstract

Heavy use of drugs impacts of the daily activities of individuals in these activities. Several groups of investigators have indeed documented changes in cognitive performance by individuals who have a long history of chronic drug use. In the case of marijuana, a wealth of information suggests that heavy long-term use of the drug may have neurobehavioral consequences in some individuals. In humans, heavy cocaine use is accompanied by neuropathological changes that might serve as substrates for cognitive dysfunctions. Similarly, methamphetamine users suffer from cognitive abnormalities that may be consequent to alterations in structures and functions. Here, we detail the evidence for these neuropsychological consequences. The review suggests that improving the care of our patients will necessarily depend on the better characterization of drug-induced cognitive phenotypes because they might inform the development of better pharmacological and behavioral interventions, with the goal of improving cognitive functions in these subsets of drug users.

## Introduction

Substance use disorders continue to be a major health concern worldwide. Chronic use of various drugs can impact brain structures and functions (1, 2). Use of these drugs may also be associated with both acute and chronic neuropsychological abnormalities (3). The present review summarizes some of the evidence documenting cognitive changes reported in drug users [with a focus on marijuana, cocaine, and methamphetamine (METH)]. We also discuss potential biological substrates for these observations. The neuropathological changes associated with the use of larger quantities of some of these drugs have been recently reviewed (1). In addition to having differential abuse liability, the use of some of these substances is also associated with differential pathoanatomic changes in the brain (1). There is also evidence that a history of substance use may also exacerbate pre-existing neuropsychological deficits (4) and comorbid neurological or psychiatric disorders (3). It is also clear that substance-related changes in neuropsychological functions may negatively impact activities of daily living, including ability to manage finances and/or holding on to jobs (5). A meta-analysis of METH users and cognition revealed that these individuals exhibited small-to-medium effect sizes for an association between neurocognitive impairment and employment (6). Cognitive domains associated with employment status included executive function, learning and memory, attention, and general intellectual ability (6). In the present review, we will discuss alterations that are linked to psychological and neural mechanisms that detect error signals and generate suitable behavioral responses (7). Also discussed is the accumulated evidence of poor learning and memory, diminished executive functions, and risky decision-making in some individuals with a history of heavy drug use (8–11).

## Marijuana Use

Marijuana is the most commonly used illicit substance (12). Investigations of cognitive functions in heavy marijuana users have recently documented poor performance in a number of cognitive subdomains. Some of these deficits appear to be related to frequency of drug use and can impact activities of daily living.

### Neuropsychological Findings

Adult marijuana users suffer from changes measured in broad cognitive domains (13, 14). These include memory (9, 13, 14, 15), attention (16), decision-making (17), and psychomotor speed (9, 18). Bolla et al. (9) reported that impairments observed in marijuana users could be measured in heavy users even after 28 days of forced abstinence during their participant stay on a closed research unit, with light use of marijuana not being associated with any significant decrements in performance (9). In a recent study, Colizzi et al. (19) studied whether functional variations in cannabinoid receptor 1 (CNR1) gene and marijuana exposure interact to modulate prefrontal functions and related behaviors. The authors suggested that deleterious effects of marijuana use may be more evident in individuals with specific genetic backgrounds that might impact receptor expression (19). Additionally, it is important to note that, even if marijuana use during early adulthood is associated with cognitive impairments in selected domains, prolonged abstinence may promote improvement in performance (13, 14, 20). These data are summarized in Table 1.

**Table 1:** **Cognitive deficits reported in marijuana users**.

Reference	Cannabis dependence	Cognitive findings
Solowij et al. (21, 22)	Adult chronic users	↓ Attention
Pope et al. (13, 14)	Adult heavy users (abstinent)	↓ Verbal memory
	Adult moderate users (abstinent)	
Bolla et al. (9)	Adult abstinent users	↓ Verbal memory
		↓ Visual memory
		↓ Executive function
		↓ Psychomotor speed
		↓ Manual dexterity
Lyons et al. (23)	Adult abstinent users	↓ General intelligence
	Twin study	
Medina et al. (24)	Adolescent abstinent users	↓ Executive function
Hanson et al. (25)	Adolescent abstinent users	↓ Verbal memory
		↓ Attention
		↓ Working memory
Battisti et al. (26)	Adult chronic users	↓ Memory recall
Griffith-Lendering et al. (27)	Adult recreational users	↓ Inhibitory control
Meier et al. (28)	Adolescent onset vs. adult onset	↓ IQ
	Prospective study	↓ Working memory
		↓ Reasoning
Solowij et al. (29)	Adolescent chronic users	↓ Decision-making, increased impulsivity
Sewell et al. (30)	Frequent and infrequent users	↓ Temporal processing in infrequent users

*↓, Cognitive deficits*.

Functional imaging studies comparing activation in both adult and adolescent chronic marijuana users to healthy controls during the performance of different cognitive tasks have reported that chronic marijuana users showed altered patterns of brain activity [Ref. (31–38), see Table 2]. There is also evidence to suggest that heavy marijuana use may produce deficits on measures of decision-making and inhibitory control that persist for long periods of time (27). Among recreational marijuana users, lack of inhibitory control depends on contextual or situational factors, with loss of control being evident only when situations or tasks involve a motivational component (27). Also, poorer cognitive performance in areas of risk-taking, decision-making, and episodic memory may influence the degree to which marijuana users engage in risky behaviors with consequent negative health consequences (39). In addition, it has been reported that the main active ingredient in marijuana, delta-9 tetrahydrocannabinol (THC), can alter time perception by impairing time estimation and production in the seconds range (30). Temporal processing changes may have functional consequences because it is relevant to many everyday tasks, including driving (30).

**Table 2:** **Functional neuroimaging studies on marijuana users performing cognitive tasks**.

Reference	Cannabis dependence	Neuroimaging method	Main findings
Block et al. (15)	Adult chronic users	PET	↓ Verbal memory
			↓ Activation in PFC
			↑ Activation in cerebellum
Bolla et al. (17)	Adult abstinent users	PET	↓ Decision-making
			↓ Activation in DLPC and OFC
			↑ Activation in cerebellum
Chang et al. (31)	Adult chronic users	fMRI	↓ Activation in cerebellum
	Adult abstinent users		Altered activation pattern in the attention network
Padula et al. (32)	Adolescent abstinent users	fMRI	↑ Activation in temporal gyrus, ACC
			↓ Activation in thalamus, pulvinar, left temporal gyrus
Tapert et al. (33)	Adolescent abstinent users	fMRI	↑ Activation in DLPC, medial frontal cortex, parietal, and occipital gyrus
Schweinsburg et al. (34)	Adolescent abstinent users	fMRI	↑ Activation in parietal cortex
			↓ Activation in DLPC and occipital cortex
Hester et al. (35)	Adult chronic users	fMRI	↓ Monitoring of interoceptive awareness
			↓ Activation in insula, ACC, parietal, and frontal cortex
Abdullaev et al. (16)	Young adult chronic users	fMRI	↓ Attention
			↑ Activation in PFC and parietal cortex
King et al. (18)	Adult chronic users	fMRI	↓ Psychomotor speed
			↓ Activation in lingual gyrus
			↑ Activation in frontal gyrus
Wesley et al. (37)	Adult chronic users	fMRI	↓ Decision-making
			↓ Activation in cerebellum, ACC, parietal, and frontal cortex
Harding et al. (38)	Adult chronic users	fMRI	↑ Functional connectivity between PFC and occipitoparietal cortex

*ACC, anterior cingulate cortex; DLPC, dorsal lateral prefrontal cortex; PFC, prefrontal cortex; ↓, decreased brain activation; ↑, increased brain activation; ↓, cognitive deficits*.

Interestingly, although much more in-depth research remains to be done on this controversial issue, marijuana use during adolescence has been reported to increase the risk of developing psychotic disorders later in life (40). THC was also reported to induce acute psychotic symptoms in healthy individuals (41) and to increase the risk of psychotic disorders after long-term use (42). A recent study by Bhattacharyya et al. (43) reported a significant relationship between the effects of THC on striatal activation, its effects on task performance, and appearance of positive psychotic symptoms, suggesting that THC might induce psychosis by influencing the neural substrate of attentional salience processing (43). Although more research is needed on this subject, there are plausible biochemical pathways that marijuana can impact to induced psychotic responses in some individuals. Specifically, the endocannabinoid system consists of cannabinoids receptors and endogenous cannabinoid ligands that interact with these receptors to impact the release of several neurotransmitters, including GABA, glutamate, and dopamine (44, 45). Therefore, it seems possible that exposure to marijuana-based psychoactive substances during adolescence could negatively impact glutamatergic and GABAergic systems, with subsequent alterations of maturation processes of these systems, resulting in psychosis-like phenomena (46). The appearance of psychiatric disturbances might also depend on the exact dose, time windows during adolescence, and/or duration of drug exposure (24, 28, 40). Interestingly, hair analyses also revealed that marijuana users with high THC concentration were more likely to exhibit schizophrenia-like symptoms (47, 48). Some of the neuroimaging and cognitive changes reported in marijuana users appear to be moderated by gender (24, 49). These findings highlight potential THC-induced neuroadaptations in the adolescent brain and support the importance of prevention and treatment of adolescent users (28). Nevertheless, this topic needs to be further investigated before any firm conclusion can be reached concerning the relationship of THC to psychosis and other psychiatric diseases.

## Cocaine Use

Although cocaine is a highly addictive agent, the vast majority of cocaine users do so recreationally over extended periods of time without developing dependence (50). Thus, documenting the potential cognitive effects of cocaine is an important public health issue because of its high prevalence in the general population. Recent neurobehavioral studies have shown that cocaine heavy users show a number of cognitive decrements that may be secondary to cocaine-induced changes in brain structure and function (1). These cognitive deficits are detailed below.

### Neuropsychological Findings

Heavy cocaine use is associated with decrements in performance in several cognitive domains [Ref. (51), detailed in Table 3]. These include problems in executive function, decision-making, increased impulsivity, abnormal visuoperception, abnormal psychomotor speed, impaired manual dexterity, poor verbal learning, and decrements in memory functions (8, 52–58). Additionally, cocaine users showed different patterns of brain activation while performing cognitive tasks [Ref. (59–67), see Table 4]. Chronic cocaine users show poor insight and judgment, lack foresight, and are also disinhibited (68). These cognitive changes are probably related to functional dysfunctions in the prefrontal cortex (69) since patients who suffer damage in this brain region manifest similar cognitive problems (70). This suggestion is supported by neuroimaging studies demonstrating hypofrontality in cocaine users performing tasks of attention and executive function (62, 71). From this perspective, the possibility that a core deficit in executive functions, such as context processing, might contribute to the well-documented impairments in top-down control that are commonly associated with heavy cocaine use (72). In addition to those observations in chronic heavy cocaine users, subtle cognitive deficits have been reported in non-dependent, recreational cocaine users (50, 73–76).

**Table 3:** **Cognitive deficits reported in cocaine users**.

Reference	Cocaine dependence	Cognitive findings
Ardila et al. (52)	Adult chronic users	↓ Verbal memory
		↓ Attention
O’Malley et al. (53)	Adult chronic users	↓ Verbal memory
		↓ Intelligence
		↓ Verbal abilities
		↓ Global neuropsychological functioning
Strickland et al. (77)	Adult abstinent users	↓ Attention
		↓ Visual memory
		↓ Psychomotor speed
Hoff et al. (54)	Adult abstinent users	↓ Spatial memory
		↓ Cognitive flexibility
		↓ Psychomotor speed
		↑ Verbal abilities
Gillen et al. (55)	Adult abstinent users	↓ Visual memory
		↑ Visual motor speed
Robinson et al. (78)	Adult chronic cocaine users	↓ Psychomotor functioning
	Adult chronic cocaine + alcohol users	↓ Global neuropsychological functioning
Bolla et al. (8)	Adult abstinent users	↓ Visuoperception
		↓ Executive function
		↓ Psychomotor speed
		↓ Manual dexterity
Aharonovich et al. (79)	Adult chronic users	↓ Attention
		↓ Memory
		↓ Spatial ability
Colzato et al. (73)	Adult recreational users	↓ Inhibitory control
Woicik et al. (80)	Adult chronic users	↓ Verbal memory
		↓ Executive function
		↓ Attention
Kalapatapu et al. (81)	Young adult chronic users	↓ Psychomotor speed
	Old adult chronic users	↓ Attention
		↓ Memory
Madoz-Gúrpide et al. (82)	Adult chronic users	↓ Executive function
Soar et al. (83)	Adult recreational users	↓ Executive function
		↓ Attention
Vonmoos et al. (84)	Adult chronic users	↓ Executive function
	Adult recreational users	↓ Attention
		↓ Working memory
		↓ Declarative memory
Winhusen et al. (68)	Adult chronic users	↓ Executive function
		↓ Inhibitory control
		↑ Apathy
Jones et al. (72)	Adult chronic users	↓ Context processing ability
Preller et al. (85)	Adult chronic users	↓ Empathy

*↓, Cognitive deficits; ↑, cognitive improvement; ↑, neurobehavioral symptoms*.

**Table 4:** **Functional neuroimaging studies on cocaine users performing cognitive tasks**.

Reference	Cocaine dependence	Neuroimaging method	Main findings
Goldstein et al. (59)	Adult chronic users	[(18)FDG PET]	↓ Visual memory
			↓ Verbal memory
			↓ Executive function
			↓ Attention
			Differential DLPC and ACC metabolism
Tucker et al. (60)	Adult abstinent users	SPECT	↓ Decision-making
			↑ Hyperperfusion in frontal cingulate and superior frontal gyrus
Kübler et al. (61)	Adult chronic users	fMRI	↓ Visuospatial working memory
			↓ Verbal working memory
			↓ Activation in prefrontal cortex, ACC, thalamus, and striatal areas
Tomasi et al. (62)	Adult chronic users	fMRI	↓ Working memory
			↓ Activation in thalamus and mesencephalon
			↑ Activation in frontal/parietal cortex
			↑ Deactivation in putamen, ACC, parahippocampal gyrus, and amygdala
Volkow et al. (86)	Adult chronic users	[(18)FDG PET]	↓ Metabolic activity in NAcc and OFC when inhibit craving
Hanlon et al. (63)	Adult chronic users	fMRI	↓ Sensorimotor abilities
			↓ Functional laterality in cortical motor areas
Moeller et al. (64)	Adult abstinent users	fMRI	↓ Activation in PFC, striatum, and thalamus
			↓ Activation in thalamus associated with poor treatment response
Volkow et al. (65)	Adult male and female chronic users	[(18)FDG PET]	↑ Brain reactivity to cocaine-cues in women
			↓ Activation in frontal, cingulate, and parietal cortex, thalamus, and midbrain in women
Camchong et al. (66)	Adult chronic users	fMRI	↓ Delay rewards
			↓ Decision-making
			↓ Learning
			Altered connectivity within the ACC network, frontal hyperconnectivity
Barrós-Loscertales et al. (67)	Adult chronic users	fMRI	↓ Activation in PFC
Preller et al. (87)	Adult chronic users	fMRI	↓ Activation in OFC

*ACC, anterior cingulate cortex; DLPC, dorsal lateral prefrontal cortex; NAcc, nucleus accumbens; OFC, orbitofrontal cortex; PFC, prefrontal cortex; ↓, decreased brain activation; ↑, increased brain activation; ↓, cognitive deficits*.

There is a compelling evidence to suggest that cocaine-associated impairments in cognitive functioning might be secondary to cocaine-induced dysfunctions in dopaminergic systems (88–93). Cerebral hypoperfusion observed in the frontal and temporo-parietal cortical areas of cocaine users (77, 94) may also subserve some of the observed cognitive deficits in these patients. These suggestions are consistent with the report of increased cerebral vascular resistance in cocaine users, abnormalities that lasted for, at least, 1 month of monitored abstinence (95).

In addition to specific deficits observed in cocaine users, these individuals may also suffer from psychosocial impairments. For example, a recent study by Preller et al. (87) suggests a relationship between social cognition test outcomes in cocaine-dependent patients and real-life social functioning. Specifically, participants showing more empathy and better mental processing abilities had a larger social network. In addition, social network size was correlated with duration and amount of cocaine use. This suggests that cocaine use and the associated altered empathy and insight may have consequences in everyday life, including fewer social contacts and deprivation of emotional support (87). Additionally, Preller et al. (85) also reported that individuals with cocaine dependence have blunted reward responses to social interactions as well as having reduced orbitofrontal cortex signals while performing a social cognition test. Taken together, these observations suggest that the treatment armamentarium may need to include interventions that boost more interactions of patients with other individuals in various social networks. This argument may explain, in part, why the affiliation-promoting peptide, oxytocin, may have beneficial effects in substance use treatment (96, 97). The possibility that social reward deficits might precede or be consequent to cocaine use needs to be investigated further (96).

In summary, although these cocaine-associated changes in cognitive functions have been well documented, their biological substrates have yet to be understood. Recent functional and structural imaging data provide ample support for impaired connectivity in frontostriatal (4, 98) and striatal-insular (99) connections that serve as neuroanatomical and functional substrates for some of the cognitive deficits reported in cocaine using individuals. A clinical approach that takes into consideration the fact that some patients may actually suffer from cognitive impairments should stimulate investigations in order to provide more details on the basic substrates of cocaine use by humans (74).

## Methamphetamine Use

Methamphetamine use is a serious public health problem (100). Long-term exposure to the drug has been shown to cause severe neurotoxic and neuropathological effects with consequent disturbances in several cognitive domains (1). These neuropsychological impairments that can impact the daily lives of METH users are detailed below.

### Neuropsychological Findings

Chronic METH users show mild signs of cognitive decline (10) affecting a broad range of cognitive functions [Ref. (5, 6, 101–112), see details in Table 5; but see also Ref. (113) for a counterargument]. A meta-analysis study by Scott et al. (107) identified significant deficits of a medium magnitude in several different cognitive processes that are dependent on the functions of frontostriatal and limbic circuits. The affected domains include episodic memory, executive functions, complex information processing speed, and psychomotor functions (107). Additionally, METH use often results in irritability, agitation, and numerous other forms of psychiatric distress probably related to the myriad of interpersonal problems experienced by these patients (114, 115). METH dependence is also associated with complaints of cognitive dysfunctions including memory problems and self-reported deficits in everyday functioning (110). Additionally, impulsive behaviors may exacerbate their psychosocial difficulties and promote maintenance of drug-seeking behaviors, especially, by those who use large amounts of the drug (116, 117). The nature and magnitude of cognitive deficits associated with chronic METH use increase the risk of poorer health outcomes, high-risk behaviors, treatment non-adherence, and repeated relapses (110, 118). These adverse consequences might be secondary to poor executive function and memory deficits that may contribute to continuous drug-seeking behaviors (70). It needs to be noted that partial recovery of neuropsychological functioning and improvement in affective distress can be achieved after a period of sustained abstinence from METH (5). Hart et al. (113) have reviewed the literature and suggested that the deficits reported may be statistically but not clinically significant. In a follow-up analysis of similar data, Dean et al. (10) came to a different conclusion. These issues are important to clinicians who are responsible for the daily and/or long-term care of patients because small deficits may be of substantial importance when it comes to patients being able to follow instructions that would help them to participate in their own care, given the high rate of recidivism in that patient population (119, 120). Therefore, identifying patients with neuropsychological deficits would allow for the development of specific cognitive or pharmacological approaches that would benefit them.

**Table 5:** **Cognitive deficits reported in methamphetamine users**.

Reference	Methamphetamine dependence	Cognitive findings
Simon et al. (101)	Adult chronic users	↓ Attention
		↓ Verbal memory
		↓ Executive function
Simon et al. (102)	Adult chronic users	↓ Psychomotor speed
		↓ Attention
		↓ Inhibitory control
Salo et al. (105)	Adult abstinent users	↓ Cognitive inhibition
Simon et al. (103)	Adult abstinent users	↓ Episodic memory
	Adult abstinent users with relapse	
	Adult chronic users	
Newton et al. (106)	Adult abstinent users	↓ Working memory
		↓ Psychomotor speed
Scott et al. (107)	Adult chronic users meta-analysis	↓ Executive function
		↓ Verbal fluency
		↓ Motor ability
		↓ Verbal memory
		↓ Language
		↓ Visuo-constructional abilities
		↓ Information processing speed
Rendell et al. (108)	Adult abstinent users	↓ Executive function
		↓ Working memory
		↓ Retro and prospective memory
Henry et al. (109)	Adult abstinent users	↓ Facial recognition
Henry et al. (110)	Adult abstinent users	↓ Functioning everyday abilities
Iudicello et al. (5)	Adult abstinent users, w or w/o relapse	↑ Global cognitive and affective improvements with sustained abstinence
	Longitudinal study	
Weber et al. (111)	Adult abstinent users	↓ Global cognitive scores = predictor of unemployment
Cattie et al. (112)	Adult abstinent users	↑ Neurobehavioral symptoms
		↓ Inhibition (self-reported)
		↓ Executive function (self-reported)

*↓, Cognitive deficits; ↑, cognitive improvement; ↑, neurobehavioral symptoms*.

Neuroimaging studies have documented several alterations in brain activation patterns induced by METH [Ref. (104, 121–128), see Table 6]. These studies reported decreased frontal activation associated with impaired decision-making (104) and cognitive control (127). Other brain regions sensitive to METH effects include the cingulate gyrus and insula (122, 128). METH users who showed impaired attention (122) and impaired cognitive control (128) exhibited abnormalities in these brain regions (see Table 6). It is worth mentioning that, in some cases, stimulant-dependent patients report clinically significant neuropsychological abnormalities prior to lifetime initiation of psychostimulant use (68).

**Table 6:** **Functional neuroimaging studies on methamphetamine users performing cognitive tasks**.

Reference	Methamphetamine dependence	Neuroimaging method	Main findings
Paulus et al. (104)	Adult abstinent users	fMRI	↓ Decision-making
			↓ Activation in PFC
Chang et al. (121)	Adult chronic users	Structural MRI	Larger globus pallidus and putamen
London et al. (122)	Adult abstinent users	[(18)FDG PET]	↓ Attention
			Differential activation in cingulate gyrus and the insula
Johanson et al. (123)	Adult abstinent users	PET	↓ Memory
			↓ Attention
			↓ Information processing speed
			↓ DAT and VMAT2 in striatal regions
Monterosso et al. (124)	Adult abstinent users	fMRI	↓ Decision-making
			↓ Cortical efficiency in frontoparietal clusters
Payer et al. (129)	Adult abstinent users	fMRI	↑ Activation in ACC
			↓ Activation in PFC
Hoffman et al. (130)	Adult abstinent users	fMRI	↑ Impulsivity
			↓ Activation in caudate, DLPC, ACC
Salo et al. (127)	Adult abstinent users	fMRI	↓ Cognitive control
			↓ Activation in PFC
Nestor et al. (128)	Adult abstinent users	fMRI	↓ Cognitive control
			↓ Activation in motor cortex/anterior cingulate gyrus, insular cortex

*ACC, anterior cingulate cortex; DLPC, dorsal lateral prefrontal cortex; PFC, prefrontal cortex; ↓, decreased brain activation; ↑, increased brain activation; ↓, cognitive deficits; ↑, cognitive improvement; ↑, neurobehavioral symptoms*.

### Recovery of Neurocognitive Functioning and Treatment Implications

Chronic use of several illicit drugs is associated with variable degrees of impaired cognitive functioning that shows different levels of improvement during sustained abstinence (3). Recovery from METH dependence is associated with improved performance in tests of mental flexibility, attention, processing speed, verbal memory, fine motor functioning, and verbal fluency (5). Improvements in performance are also seen in abstinent marijuana users (13, 14). Moreover, Brewer et al. (131) found that activation in corticostriatal regions, linked to cognitive control, correlated with abstinence and cocaine-free urine toxicology (131). There was also an inverse correlation between prefrontal cortex activation and treatment retention (131), thus supporting the notion that identification of patients with cognitive deficits are important for the long-term care of these patients (3, 132). This suggestion is supported by the results of a very recent report that strength of craving for METH can be reduced by cognitive strategies (133). In addition, patients who participated in computer-assisted cognitive behavioral therapy showed improved task performance and reduced task-related signal changes in several regions implicated in cognitive control, impulse control, and motivational salience, including the anterior cingulate and midbrain (134).

## Conclusion

Chronic use of illicit substances, including marijuana, cocaine, and METH, is associated with abnormal goal-directed behaviors that are thought to be the manifestations of altered cortico-striatal-limbic circuits (2, 135). Nevertheless, the wealth of clinical presentations, neuroimaging studies, and some pathological findings suggest that the biochemical and structural effects of chronic heavy use of drugs may reach beyond the boundaries of these reward circuits (1). The data reviewed here indicate that chronic use of illicit drugs is accompanied by moderate cognitive impairments in some patients. These observations may be related to functional and structural changes in various brain regions, including both cortical and subcortical regions of the human brain (1, 98, 136). In addition, it has been reported frontal deficits in psychostimulant-dependent patients reporting current clinically neurobehavioral abnormalities may be linked to pre-existing abnormalities (68). Because drug dependence develop over many months, it is likely that drug-related changes of behaviors may be modulated by some of these pathological phenomena in such a way as to significantly impact the clinical course of chronic use of these substances. Thus, impaired learning and memory functions might negatively impact the ability of a specific subset of patients to benefit from general treatment approaches. This inability may explain, in part, the high rate of recidivism in this patient population. This argument suggests that approaches to these individuals should take into consideration the diversity of patterns of substance use and clinical presentations. This argument suggests that thorough neuropsychological and neuroimaging assessments should be undertaken to identify these subsets of drug users. This approach should help to dichotomize patients as being unimpaired or impaired, with specific cognitive and pharmacological treatments targeting subgroups of patients. Present approaches that group all patients together need to be revamped to allow for more rational and data-driven approaches to treatment.

## Conflict of Interest Statement

The authors declare that the research was conducted in the absence of any commercial or financial relationships that could be construed as a potential conflict of interest.
